# Occupational disease risks in natural resource-based industries among a large cohort of workers in Ontario, Canada

**DOI:** 10.1177/10519815261476663

**Published:** 2026-08-21

**Authors:** Taryn Thompson, Katie Goggins, Paul A Demers, Katherine J Jardine, Meg Parker, Jeavana Sritharan

**Affiliations:** 1 Occupational Cancer Research Centre, 573450 Ontario Health, Toronto, ON, Canada; 2 Dalla Lana School of Public Health, 274071 University of Toronto, Toronto, ON, Canada; 3 Centre for Research in Occupational Safety and Health, 7728 Laurentian University, Sudbury, ON, Canada; 4 Workplace Safety North, Sudbury, ON, Canada

**Keywords:** carpal tunnel syndrome, extraction and processing industry, forestry, lung diseases, myocardial infarction, neoplasms, occupational diseases, raynaud disease

## Abstract

**Background:**

Natural resource-based industries are critical to many economies. Although workers in these industries face several hazardous exposures, the industry-specific risks of occupational diseases remain underexplored.

**Objective:**

This study aimed to identify high-risk occupational diseases among workers in Ontario's natural resource-based industries.

**Methods:**

Workers were identified in the Occupational Disease Surveillance System (ODSS), a system linking workers’ compensation data (1983–2019) to health records. Cox proportional hazard models were used to estimate hazard ratios (HR) and 95% confidence intervals (CI) for diagnoses of various occupational diseases, adjusted for age at start of follow-up, birth year, and sex.

**Results:**

Overall, 10,286 workers were identified in forestry and logging, 40,122 in wood products, 25,828 in pulp and paper, and 24,921 in mining. Elevated risks of specific cancers, such as lung, and laryngeal cancer, were observed among most workers in the four natural resource-based industries. Colorectal cancer risks were higher among pulp and paper, and mining workers. Elevated risks of non-cancer outcomes, including carpal tunnel syndrome, acute myocardial infarction, idiopathic pulmonary fibrosis, and chronic obstructive pulmonary disease were also observed. For some outcomes, such as silicosis, where case counts were low, an elevated risk was only observed among metal miners.

**Conclusions:**

These findings demonstrate that workers in natural resource-based industries remain at high risk for several occupational diseases, highlighting the critical need for industry-specific early and targeted prevention initiatives.

## Introduction

Natural resource-based industries are critical to many economies but involve hazardous working conditions that pose serious health risks to workers. Common physical hazards in the natural resource-based industries may include exposure to noise, vibration, and radiation.^1–4^ Previously identified chemical hazards in mining include respirable crystalline silica (RCS), asbestos, coal dust, diesel engine exhaust, and metals.^1,5^ The wood products, and forestry and logging industries are primarily affected by wood dust, diesel particulate exposures, and biological hazards (e.g., fungal spores, moulds, bacteria, and bioaerosols).^4,6^ Although less documented in research, the pulp and paper industry also involves significant exposures, including asbestos, carbon monoxide, formaldehyde, nitrogen dioxide, dusts, sulphuric acid, solvents, diphenyl, polychlorobiphenyls, sulphur dioxide, hydrogen sulphide, and mercaptans and biological hazards.^7^ Despite improvements in occupational safety, there remains a lack of comprehensive surveillance and standardized reporting across these industries.

In 2025, the Workplace Safety and Insurance Board (WSIB) in Ontario reported the following distribution of allowed occupational disease categories: acute exposures and effects (56%), noise-induced hearing loss (26%), chronic exposures and effects (8%), COVID-19 related (7%), long-latency illnesses (1%), and uncoded occupational diseases (1%).^8^ Although long-latency illnesses only accounted for 1% of allowed occupational disease by count, they represented 44% of the annual cost in 2024 and 17% in 2025.^8^

Active occupational disease surveillance systems include Occupational Disease Surveillance System (ODSS) (Ontario, Canada),^9^ MALPROF (Italy),^10^ and The Health and Occupational Research Network (THOR) (United Kingdom).^11^ Some surveillance systems have tried to amalgamate their chronic disease data but have encountered challenges. For instance, Canadian health insurance registries are managed at the provincial and territorial levels, resulting in discrepancies in reporting metrics. However, a harmonized system has been suggested to improve national surveillance initiatives.^12^ For example, Monitoring Trends in Occupational Diseases and Tracing New and Emerging Risks in a Network (Modernet) involves 14 European countries providing information from their chronic disease systems.^13^ Another study used the Delphi technique to gather expert consensus on surveillance elements to inform a tailored evaluation framework.^14^ The major themes regarding current occupational health and safety were resources and feasibility, data collection, flexibility, and the interrelatedness among elements.

Ideally, occupational disease surveillance systems are used to: (1) identify trends (i.e., emerging risks and patterns in diseases), (2) inform prevention efforts (i.e., policies, regulations, and training development), (3) support intervention evaluation and research, and ultimately (4) protect the occupational health and safety of the worker. The ODSS has been successfully used to identify associations between work and disease in Ontario, including cancers (e.g., lung, breast) and non-malignant diseases (e.g., asthma, carpal tunnel syndrome (CTS)).^15–18^ It has also been used to identify sex differences across a range of occupational diseases.^15,16^ While previous efforts have fulfilled step 1 of identifying trends, this research uses the ODSS to identify occupational disease risks in specific industries to better inform targeted prevention efforts.

The risks of many occupational diseases have not been well characterized within specific natural resource-based industries. Among the studies that have estimated disease risks in these sectors, few have used a cohort large enough to examine risk at a granular industry level. Moreover, because workplace exposures vary by region and industry, a cohort composed of local workers provides a more accurate reflection of disease risk in these specific industries in Ontario. The objective of this study was to identify high-risk occupational diseases in natural resource-based industries, specifically forestry and logging, wood products, pulp and paper, and mining industries, using a large cohort of workers in Ontario, Canada.

## Methods

### Study cohort

The ODSS monitors occupational diseases among 2.36 million Ontario workers. The ODSS was created using accepted lost time compensation claims records from the WSIB from 1983 to 2019.^19,20^ The WSIB records contain valuable information on worker characteristics and corresponding occupation and industry records related to the accepted claims. Workers were matched to records in the Registered Persons’ Database (RPDB), using deterministic (i.e., full name, sex, birth date) and probabilistic (i.e., partial names or incomplete information) linkage methods (Figure 1). The RPDB provides information on death (where applicable), emigration out of Ontario, and a unique Health Insurance Number (HIN) for all Ontarians registered for provincial health insurance.^20^ Workers were then linked to cancer and other health records to identify the occupational diseases of interest through both deterministic linkage (using HIN) and probabilistic linkage (without HIN, using name, sex, birth date, and death date). Workers with missing data were excluded.

**Figure 1.**
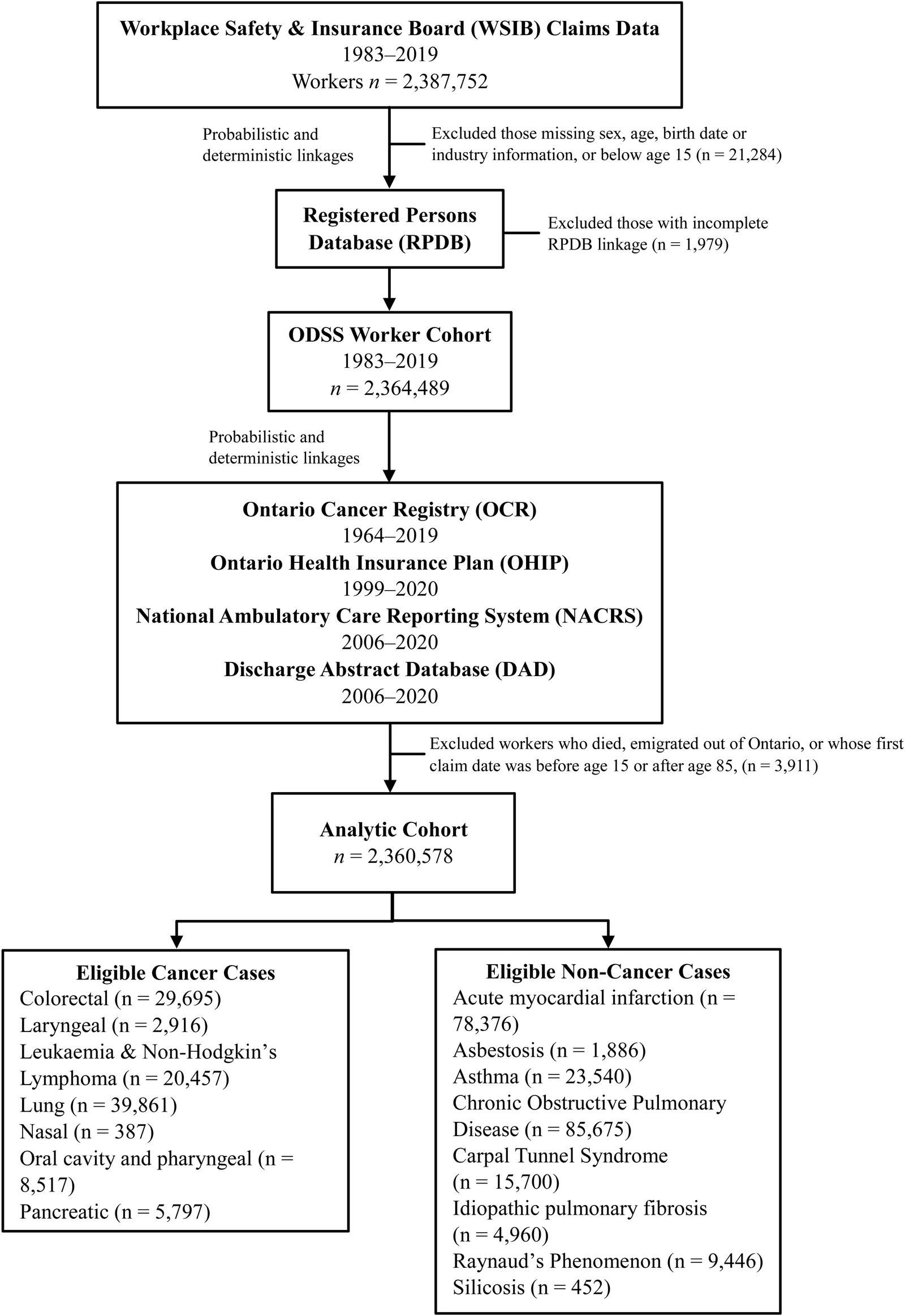
Flow chart describing the Occupational Disease Surveillance System linkage process and the identification of occupational diseases in the cohort.

### Industry classification

Industry information was obtained from WSIB records for each worker in the ODSS. Industry was coded using the 1970 and 1980 Standard Industrial Classification (SIC) systems. Using these systems, industry was classified into three levels: division (broadest), major (intermediate), and minor (specific) groups.

This paper focuses on the major- and minor-level natural resource-based industries, specifically forestry and logging, wood products, pulp and paper, and mining industries, which are most prevalent in Northern Ontario. The forestry industry includes logging and forestry services. The wood products industries include sawmills, planning and shingle mills, veneer and plywood mills, sash, door and other millwork plants, wooden box and pallet operations, coffin and casket industry, and miscellaneous wood products industries. The pulp and paper industry includes pulp and paper mills, paper box and bag manufacturers, and miscellaneous paper converters. The mining industry includes metal mines (gold, uranium, iron, and miscellaneous metal mines), non-metal mines (peat industry, crude petroleum and natural gas industry, asbestos, gypsum, non-metal, and coal mines), quarries and sandpits (stone quarries, and sandpits or quarries), and services incidental to mining (contract drilling industry, and miscellaneous services). In the case that a worker had multiple accepted lost time claims while employed in different industries, they were included in each respective industry analysis. However, this represents a relatively small subset of workers and has not been shown to meaningfully impact the results.

### Case ascertainment

The occupational diseases examined in this study included leukaemia and non-Hodgkin's lymphoma (NHL), colorectal, laryngeal, lung, oral cavity and pharyngeal, pancreatic, and nasal cancer, acute myocardial infarction (AMI), asbestosis, asthma, CTS, chronic obstructive pulmonary disease (COPD), idiopathic pulmonary fibrosis (IPF), Raynaud's phenomenon (RP), and silicosis. For cancer outcomes, workers were linked to the Ontario Cancer Registry (1964–2019). Cancer diagnoses were coded using the International Classification of Diseases for Oncology 3^rd^ Edition. For other non-cancer outcomes, workers were linked to the Ontario Health Insurance Plan (OHIP) (1999–2020), National Ambulatory Care Reporting System (NACRS) (2006–2020), and the Discharge Abstract Database (DAD) (2006–2020) to identify physician billing records, emergency department visits, and hospitalizations, respectively. These other non-cancer outcomes were coded using the International Classification of Diseases 9^th^ Revision (ICD-9) and 10^th^ Revision (ICD-10) codes (Supplemental Table 1). The follow-up period differs between cancer and non-cancer outcomes as cancer registry data extends back further than physician billing and hospital records.

### Statistical analysis

Workers were followed from the date of their first accepted WSIB claim to either their first diagnosis of a health outcome, age 85 years, emigration out of Ontario, death, or end of the follow-up period (December 31, 2019, for cancer; December 31, 2020, for non-cancer outcomes). Cox proportional hazard regression models were used to estimate the hazard ratios (HR) and 95% confidence intervals (CI) for each occupational disease in the specific industries, compared to all other workers in the ODSS. Models were adjusted for age at the start of follow-up, birth year, and sex. Case counts less than six were not reported in accordance with organizational reporting policies. All statistical analysis was conducted in R (version 4.4.2) and SAS (V.9.4).

## Results

In the overall cohort, 10,286 workers were identified in the forestry and logging industry, 40,122 in the wood products industry, 25,828 in the pulp and paper industry, and 24,921 in the mining industry (Table 1). The characteristics of the overall cohort were similar to those of the four industries. However, the overall cohort had a higher proportion of female workers (35.4%), compared to the forestry and logging (4.5%), wood products (11.1%), pulp and paper (20.6%), and mining (2.7%) industries, all of which are male dominated. Workers in each specified industry group are compared to all other workers in all other industry groups.

**Table 1.** Characteristics of workers in selected natural resource-based industries and the overall ODSS cohort.

	Forestry & logging industry (n = 10,286)	Pulp & paper industry (n = 25,828)	Wood products industry (n = 40,122)	Mining industry (n = 24,921)	Overall ODSS cohort(n = 2,360,578)
Sex					
Female	460 (4.5%)	5314 (20.6%)	4446 (11.1%)	664 (2.7%)	835,446 (35.4%)
Male	9826 (95.5%)	20,514 (79.4%)	35,676 (88.9%)	24,257 (97.3%)	1,525,132 (64.6%)
Birth year					
Mean (sd)	1955 (13.9)	1955 (13.6)	1961 (13.9)	1952 (15.2)	1960 (15.7)
Median [Min, Max]	1956 [1908, 1999]	1956 [1901, 2002]	1962 [1903, 2002]	1953 [1901, 2004]	1960 [1898, 2004]
Years of follow-up					
Mean (sd)	27.5 (9.1)	27.0 (8.9)	25.3 (9.7)	26.3 (9.7)	22.9 (10.5)
Median [Min, Max]	30.5 [0.09, 38.0]	29.6 [0.04, 38.0]	27.6 [0.008, 38.0]	29.3 [0.02, 38.0]	24.6 [0.003, 38.0]
Age at start of follow-up					
Mean (sd)	34.4 (12.0)	35.8 (12.2)	31.7 (11.7)	37.8 (12.4)	35.7 (12.6)
Median [Min, Max]	32.0 [15.0, 77.0]	34.0 [15.0, 83.0]	29.0 [15.0, 81.0]	36.0 [15.0, 84.0]	34.0 [15.0, 84.0]
Age at end of follow-up					
Mean (sd)	61.9 (13.0)	62.8 (12.3)	57.1 (13.0)	64.1 (13.2)	58.6 (14.5)
Median [Min, Max]	62.0 [19.0, 85.0]	62.0 [18.0, 85.0]	57.0 [18.0, 85.0]	65.0 [16.0, 85.0]	59.0 [15.0, 85.0]

sd = standard deviation, ODSS = Occupational Disease Surveillance System.

### Forestry and logging, wood products, and pulp and paper industries

Among forestry and logging workers, the risks of IPF, CTS, COPD, AMI, lung, and laryngeal cancer were over 1.2 times higher compared to all other workers in the cohort, adjusted for birth year, sex, and age at the start of follow-up (Table 2). A decreased risk of asthma was observed among forestry and logging workers. Workers employed in wood products industries experienced greater risks of COPD, AMI, lung, and nasal cancer. The risk of asbestosis was also lower among these workers. When examining the six minor-level groups that compose the wood products industry, higher risks of CTS (HR 2.33, 95% CI 1.41–3.87), RP (HR 2.15, 95% CI 1.16–4.00), IPF (HR 1.76, 95% CI 0.84–3.69), and leukaemia and non-Hodgkin's lymphoma (HR 1.56, 95% CI 1.06–2.29) were observed among veneer and plywood workers (Supplemental Table 2). Further, the risks of oral cavity and pharyngeal cancer (HR 1.62, 95% CI 1.00–2.60), colorectal cancer (HR 1.34, 95% CI 0.98–1.82), and COPD (HR 1.45, 95% CI 1.24–1.70) were elevated for wooden box and pallet operations workers. The risk of IPF was notably increased among workers in miscellaneous wood products industries (HR 1.57, 95% CI 0.97–2.53), and decreased in sash, door and other millwork plants (HR 0.52, 95% CI 0.31–0.86). Elevated and reduced risks of pancreatic cancer were observed across multiple minor-level wood products industries. However, the estimates were statistically imprecise.

**Table 2.** Risk of occupational diseases among workers in forestry and logging, wood products, and pulp and paper industries, compared to all other workers in the ODSS cohort.

Occupational disease	Forestry & logging		Wood products		Pulp & paper	
	Cases (Workers)	HR* (95% CI)	Cases (Workers)	HR* (95% CI)	Cases (Workers)	HR* (95% CI)
Cancer						
Oral cavity and pharyngeal	50 (10,277)	1.00 (0.76–1.32)	143 (40,075)	1.05 (0.89–1.23)	104 (25,809)	0.93 (0.77–1.13)
Colorectal	145 (10,278)	0.93 (0.79–1.10)	418 (40,073)	1.01 (0.92–1.12)	427 (25,809)	1.11 (1.01–1.22)
Pancreatic	31 (10,283)	1.01 (0.71–1.44)	79 (40,088)	0.97 (0.77–1.21)	73 (25,818)	0.95 (0.76–1.20)
Laryngeal	23 (10,282)	1.22 (0.81–1.84)	49 (40,086)	1.07 (0.81–1.43)	44 (25,815)	1.06 (0.79–1.43)
Lung	254 (10,277)	1.20 (1.06–1.36)	586 (40,078)	1.08 (1.00–1.17)	551 (25,812)	1.03 (0.94–1.12)
Leukaemia and non-Hodgkin's lymphoma	99 (10,277)	0.97 (0.79–1.18)	262 (40,067)	0.91(0.81–1.03)	245 (25,804)	0.98 (0.86–1.11)
Nasal	< 6 (10,283)	−	8 (40,085)	1.29 (0.64–2.61)	6 (25,818)	1.12 (0.50–2.51)
Non-cancer						
Carpal tunnel syndrome	37 (1367)	1.43 (1.04–1.98)	162 (9511)	1.08 (0.92–1.26)	95 (3488)	1.38 (1.12–1.68)
Acute myocardial infarction	492 (6987)	1.30 (1.19–1.42)	1509 (32,622)	1.13 (1.08–1.19)	1140 (19,635)	1.11 (1.05–1.18)
Raynaud's phenomenon	16 (1395)	0.74 (0.46–1.21)	110 (9750)	1.01 (0.84–1.22)	59 (3622)	1.14 (0.88–1.47)
Chronic obstructive pulmonary disease	501 (6851)	1.35 (1.24–1.48)	1613 (32,184)	1.21 (1.15–1.27)	1249 (19,326)	1.15 (1.09–1.22)
Asthma	35 (1327)	0.76 (0.54–1.06)	243 (9077)	0.89 (0.78–1.01)	117 (3381)	1.02 (0.85–1.23)
Asbestosis	< 6 (7087)	−	18 (32,894)	0.57 (0.36–0.91)	33 (19,895)	1.27 (0.90–1.79)
Idiopathic pulmonary fibrosis	34 (7090)	1.57 (1.12–2.20)	66 (32,905)	0.99 (0.78–1.27)	81 (19,912)	1.31 (1.05–1.64)

HR*=hazard ratio, adjusted for birth year, sex, and age at start of follow up; CI = confidence interval; ODSS = Occupational Disease Surveillance System.

Elevated risks of CTS, IPF, asbestosis, COPD, RP, AMI, colorectal, and nasal cancer were observed among workers in the pulp and paper industry compared to all other workers in the ODSS, adjusted for birth year, sex, and age at the start of follow-up. The risks of these diseases were relatively similar across the three minor-level pulp and paper industries (i.e., pulp and paper mills, paper box and bag manufacturers, and miscellaneous paper converters) (Supplemental Table 3). Among the three minor-level industries, pulp and paper mill workers had a significantly higher risk of CTS (HR 2.66, 95% CI 1.86–3.81). Those working in pulp and paper mills were also the only pulp and paper group to have an elevated risk of pancreatic cancer (HR 1.21, 95% CI 0.85–1.73). In contrast, paper box and bag manufacturers had a reduced risk of pancreatic cancer (HR 0.66, 95% CI 0.42–1.05). Paper box and bag manufacturers also had a higher risk of asthma (HR 1.26, 95% CI 0.99–1.62), which was not observed in any other group examined in this study. Additionally, there is some evidence of increased risks of laryngeal (HR 1.36, 95% CI 0.82–2.25) and lung cancer (HR 1.11, 95% CI 0.96–1.28) among workers in the miscellaneous paper converters industry. Miscellaneous paper converters (HR 1.61, 95% CI 0.91–2.83), and pulp and paper mill workers (HR 1.41, 95% CI 0.80–2.48) also experienced higher risks of asbestosis.

### Mining industry

Table 3 presents elevated risks of several cancers among those in the mining industry compared to all other workers in the ODSS, adjusted for birth year, sex, and age at the start of follow-up.

**Table 3.** Risk of occupational diseases among workers in major mining industries, compared to all other workers in the ODSS cohort.

Occupational disease	Metal mines		Non-metal mines		Quarries & sandpits		Services incidental to mining	
	Cases (Workers)	HR* (95% CI)	Cases (Workers)	HR* (95% CI)	Cases (Workers)	HR* (95% CI)	Cases (Workers)	HR* (95% CI)
Cancer								
Oral cavity and pharyngeal	103 (14,258)	1.16 (0.95–1.41)	14 (2064)	1.33 (0.79–2.25)	17 (4226)	0.99 (0.62–1.59)	18 (4770)	0.89 (0.56–1.41)
Colorectal	353 (14,247)	1.15 (1.03–1.27)	44 (2063)	1.24 (0.92–1.66)	69 (4223)	1.30 (1.02–1.64)	58 (4767)	0.96 (0.74–1.24)
Pancreatic	85 (14,270)	1.44 (1.16–1.78)	6 (2065)	0.86 (0.39–1.92)	13 (4228)	1.25 (0.72–2.15)	9 (4771)	0.76 (0.39–1.46)
Laryngeal	48 (14,260)	1.25 (0.94–1.66)	<6 (2065)	−	17 (4226)	2.75 (1.71–4.44)	10 (4772)	1.38 (0.74–2.56)
Lung	581 (14,226)	1.34 (1.23–1.45)	53 (2062)	1.06 (0.81–1.39)	128 (4227)	1.81 (1.52–2.15)	97 (4768)	1.21 (0.99–1.48)
Leukaemia and non-Hodgkin's lymphoma	246 (14,257)	1.32 (1.16–1.50)	23 (2064)	1.02 (0.68–1.54)	41 (4222)	1.16 (0.86–1.58)	38 (4769)	0.93 (0.68–1.28)
Nasal	6 (14,267)	1.51 (0.67–3.40)	<6 (2066)	−	<6 (4228)	−	<6 (4772)	−
Non-cancer								
Carpal tunnel syndrome	43 (1685)	1.71 (1.27–2.31)	7 (360)	1.25 (0.60–2.62)	27 (1066)	1.74 (1.19–2.53)	13 (864)	1.06 (0.62–1.83)
Acute myocardial infarction	895 (10,258)	1.25 (1.17–1.33)	92 (1603)	0.98 (0.79–1.20)	212 (3436)	1.33 (1.16–1.52)	171 (3166)	1.14 (0.98–1.33)
Raynaud's phenomenon	32 (1824)	1.36 (0.96–1.92)	10 (374)	2.00 (1.07–3.72)	10 (1080)	0.79 (0.42–1.46)	13 (894)	1.36 (0.79–2.34)
Chronic obstructive pulmonary disease	969 (9904)	1.37 (1.29–1.46)	125 (1577)	1.32 (1.11–1.57)	256 (3364)	1.68 (1.49–1.90)	266 (3117)	1.87 (1.66–2.11)
Asthma	38 (1751)	0.85 (0.62–1.17)	<6 (353)	−	27 (1017)	0.96 (0.66–1.40)	13 (863)	0.57 (0.33–0.97)
Asbestosis	19 (10,437)	0.88 (0.56–1.38)	7 (1626)	2.72 (1.29–5.71)	<6 (3482)	−	<6 (3205)	−
Silicosis	20 (10,403)	5.35 (3.40–8.43)	<6 (1626)	−	<6 (3481)	−	6 (3199)	7.89(3.52–17.69)
Idiopathic pulmonary fibrosis	83 (10,445)	1.71 (1.38–2.13)	9 (1629)	1.53 (0.79–2.94)	15 (3483)	1.74 (1.05–2.88)	15 (3206)	1.91 (1.15–3.18)

HR*, hazard ratio. Adjusted for birth year, sex, and age at start of follow up; CI, confidence interval; ODSS, occupational disease surveillance system.

These risks were notably higher among quarry and sandpit workers, specifically those employed in stone quarries, who were at the greatest risk of laryngeal (HR 3.48, 95% CI 1.66–7.30), lung (HR 2.14, 95% CI 1.62–2.83), and colorectal cancer (HR 1.46, 95% CI 0.99–2.15). The risk of pancreatic cancer was also elevated among stone quarry workers (HR 1.71, 95% CI 0.77–3.82). Similarly, those in the metal mining industry had particularly increased risks of pancreatic cancer, and leukaemia and NHL. Among the four specific groups in the metal mining industry, uranium miners had the highest risk of pancreatic (HR 1.84, 95% CI 1.11–3.05) and lung cancer (HR 1.84, 95% 1.52–2.23). Gold miners also largely contributed to the increased lung cancer risk in the metal mining industry (HR 1.41, 95% CI 1.16–1.71). The specific miscellaneous metal mine industry exhibited higher risks of leukaemia and NHL (HR 1.39, 95% CI 1.20–1.61), colorectal (HR 1.22, 95% 1.08–1.38), pancreatic (HR 1.39, 95% CI 1.07–1.81), laryngeal (HR 1.26, 95% CI 0.89–1.78), lung (HR 1.24, 95% CI 1.12–1.38), and nasal cancer (HR 2.24, 95% CI 1.00–5.04).

Among workers employed in the major-level industry of services incidental to mining, the elevated risk of lung cancer was driven by miscellaneous services (HR 1.30, 95% CI 1.03–1.65). Further, only those in the specific non-metal mining industry had an elevated risk of oral cavity and pharyngeal cancer (HR 2.05, 95% CI 1.07–3.94). This group also experienced an elevated risk of leukaemia and NHL (HR 1.41, 95% CI 0.82–2.44). Some evidence was observed that workers in the crude petroleum and natural gas industry, a minor-level of non-metal industries, had an elevated risk of colorectal cancer (HR 1.35, 95% CI 0.91–2.00).

The risk of silicosis was substantially higher among those employed in the metal mining industry and services incidental to mining compared to all others in the ODSS cohort. The increased risk in the metal mining industry was driven by gold (HR 15.73, 95% CI 8.11–30.53) and uranium miners (HR 5.77, 95% CI 1.85–17.98). Those working in the miscellaneous metal mining industry also had a higher risk of silicosis (HR 2.81, 95% CI 1.39–5.67). Additionally, the risk of asbestosis was elevated among non-metal miners, which included a small number of asbestos miners. Those employed in the crude petroleum and natural gas industry also contributed to higher risks of asbestosis among non-metal mining workers (HR 3.87, 95% 1.61–9.30). Elevated risks were observed among miners for other non-cancer occupational diseases, such as AMI, COPD, CTS, IPF, and RP. These findings were consistent across most major- and minor-level mining industries. Among those in the metal mining industry, gold and uranium miners had higher risks of AMI (gold: HR 1.56, 95% CI 1.35–1.82; uranium: HR 1.60, 95% CI 1.37–1.87), COPD (gold: HR 1.67, 95% CI 1.44–1.94; uranium: HR 1.72, 95% CI 1.48–2.00), and IPF (gold: HR 3.08, 95% CI 1.98–4.78; uranium: HR 2.23, 95% CI 1.30–3.85). The risks of CTS (HR 1.70, 95% CI 1.22–2.36) and these outcomes were also elevated among miscellaneous metal miners (AMI: HR 1.12, 95% CI 1.03–1.21; COPD: HR 1.24, 95% CI 1.15–1.34; IPF: HR 1.37, 95% CI 1.03–1.81). Gold miners also had higher risks of RP (HR 2.59, 95% CI 1.29–5.17) and CTS (HR 1.55, 95% CI 0.70–3.45). Of the six specific non-metal mining groups, the risk of IPF was notably higher among workers in the crude petroleum and natural gas industry (HR 2.61, 95% CI 1.30–5.21). Workers in the specific non-metal mining industry had elevated risks of COPD (HR 1.36, 95% CI 1.04–1.78) and RP (HR 2.69, 95% CI 1.21–5.99). Gypsum mining industry workers also had a higher risk of COPD (HR 1.84, 95% CI 0.88–3.87).

The major-level industry of quarries and sandpits was further divided into stone quarries, and sandpits or quarries. Workers in both minor level industries experienced elevated risks of most non-cancer outcomes examined in this study, including CTS (stone: HR 1.53, 95% CI 0.82–2.85; sandpits: HR 1.87, 95% CI 1.16–3.01), COPD (stone: HR 1.86, 95% CI 1.53–2.25; sandpits: HR 1.62, 95% CI 1.39–1.89), IPF (stone: HR 2.22, 95% CI 1.06–4.67; sandpits: HR 1.41, 95% CI 0.70–2.81), and AMI (stone: HR 1.23, 95% CI 0.98–1.54; sandpits: HR 1.36, 95% CI 1.16–1.61). Of the two minor-level groups within services incidental to mining, those working in the contract drilling industry had elevated risks of CTS (HR 2.35, 95% CI 1.06–5.23) and COPD (HR 2.15, 95% CI 1.77–2.62). Workers employed in miscellaneous services also experienced higher risks of COPD (HR 1.75, 95% CI 1.50–2.03), as well as IPF (HR 2.29, 95% CI 1.33–3.94), AMI (HR 1.17, 95% CI 0.98–1.40), and RP (HR 1.42, 95% CI 0.79–2.57). Finally, the risk of asthma was lower among workers across all mining industries. Workers in services incidental to mining had a particularly low risk of asthma.

## Discussion

This study identified elevated risks of several occupational diseases among workers in the natural resource-based industries. Workers in these four industries exhibited increased risks of laryngeal and lung cancer. Colorectal cancer risk was elevated among most miners and pulp and paper workers. The risks of leukaemia and NHL, oral cavity and pharyngeal, pancreatic, and nasal cancer were only higher among certain major-level mining industries. Elevated risks of non-cancer outcomes, including IPF, COPD, CTS, and AMI, were generally observed across all four industries, except for silicosis, which was elevated only among miners. These findings were anticipated for certain diseases, as workers in these sectors are commonly exposed to occupational hazards known to contribute to adverse health outcomes, such as dusts and vibration.^1–4,21,22^

### Lung cancer

Approximately 15% of lung cancer cases are linked to workplace exposures in Canada.^23^ These exposures include diesel engine exhaust from heavy equipment used in the forestry and logging, and mining industries, RCS exposure from mines and quarries,^1,24^ nickel from mining and processing,^24,25^ and radon from both uranium and possibly non-uranium mines.^1,24,26^ Additionally, asbestos is a known carcinogen that has been linked to several cancers, including lung cancer.^24^ Canada's asbestos mines closed in 2012, and Ontario's last asbestos mine closed in the 1970's,^27^ but it may still be present in some mines and pulp and paper mills.^3^

Elevated risks of lung cancer were observed among workers in the forestry and logging, wood products, and mining industries, with the strongest associations observed in the metal mining, quarries and sandpits, and sawmills, planning and shingle industries. These findings are generally consistent with prior studies exploring lung cancer risks in these industries and among similar occupations.^28–30^ A previous study using the Nordic Occupational Cancer surveillance system found an elevated smoking-adjusted risk of lung cancer among female wood workers, and male miners and quarry workers.^29^ Finnish male forestry and wood workers also had a higher risk of lung cancer; however, this increase was not observed in other Nordic countries.^29^ These mixed results may suggest regional differences in workplace practices, types, or magnitudes of exposures.

This study could not account for cigarette smoking, which is strongly associated with lung cancer.^24^ However, previous studies that have controlled directly or indirectly for smoking still observed an association between occupational exposures common to natural resource-based industries (e.g., nickel, RCS) and lung cancer risk.^16,30–32^

### Other cancer risks

Less is known about the causes of laryngeal cancer. However, the International Agency for Research on Cancer (IARC) has identified asbestos and strong inorganic acid mists as associated exposures.^24^ This study observed significantly increased risks of laryngeal cancer among quarry and sandpit workers, as well as elevated risks among forestry and logging, metal mining, and services incidental to mining. These findings were supported by previous research that observed an association between this cancer and employment as a miner, quarryman, or forestry worker.^33,34^ Lifestyle factors such as alcohol, smoking, and second-hand smoke, which were not available in this study, may contribute to risk of laryngeal cancer.^24^ However, a 2004 case-control study found that adjusting for occupational exposures significantly reduced alcohol- and tobacco-related risks, indicating workplace hazards may also play a role.^35^

This study also observed elevated risks of colorectal cancer among workers in the pulp and paper, and mining industries. IARC has suggested that asbestos, a potential carcinogenic exposure in both industries, may be associated with colorectal cancer.^1,3,24^ Further, a recent meta-analysis of 82 studies observed higher risks of this cancer among asbestos-exposed workers, with a slight but statistically imprecise increase for mining occupations.^36^ A previous study of Canadian workers also observed some evidence that high diesel engine exhaust exposures could be linked to colorectal cancer.^37^

Miners, specifically those employed in metal mines, were also at higher risks of leukaemia and NHL, pancreatic, and nasal cancer. Conflicting findings have been observed from the few studies that examined leukaemia and NHL incidence and mortality in the Canadian mining industry.^28,38,39^ Pancreatic cancer risk among miners also remains understudied. However, a previous hospital-based case-control study examining pancreatic cancer by job title observed that pancreatic cancer diagnosis was associated with mining and stone cutting professions.^40^ IARC also classified radiation, an occupational hazard in underground mining environments, as a possible risk factor for leukaemia and NHL, and pancreatic cancer.^1,24^ The elevated risk of nasal cancer among metal miners was driven by workers employed in nickel mines, which were included in the miscellaneous metal mining industry. This finding aligns with previous research as nickel compounds, such as those present in nickel refineries, have been identified as carcinogens linked to nasal cancer.^24^

### Chronic obstructive pulmonary disease

The American Thoracic Society and European Respiratory Society estimate that 14% of all COPD cases (31% for never smokers) are linked to workplace exposures to dusts, fibres, and fumes.^41^ This study observed significantly elevated risks of COPD across all major-level industries. There is mixed evidence of higher COPD risks among forestry and logging workers.^42–44^ A study using Health and Retirement Study data did not observe an association between the agriculture, forestry and fishing industry or occupational groups, and COPD risk.^43^ However, the current study examined the forestry and logging industry separately from other groups, and at a granular level, allowing for more detailed and precise risk estimates. Further, forestry and logging workers are exposed to multiple substances that are associated with poor respiratory health, including diesel engine exhaust, pesticides, and organic dusts.^6,42,45^ Results from the National Health Interview Survey in the United States also indicated that male workers in the agriculture, forestry, fishing and hunting industries had the highest COPD prevalence compared to other occupation groups captured by the survey.^44^ Wood product workers experience similar exposures to forestry and logging workers and also experienced an elevated risk of COPD.^4^ These findings are consistent with previous studies that observed an increased risk of COPD among sawmill workers in British Columbia.^46,47^ Pulp and paper workers also exhibited increased risks of COPD, which may be associated with dust and asbestos exposure.^3,42^ Previous studies have examined paper dust exposure as a risk factor for COPD; however, the findings are conflicting.^42,43^ Notably, COPD is underdiagnosed in Canada, and thus the findings of this study may underestimate the true risk among workers in these key natural resource-based industries.^48^

Workers in the mining industries were also notably impacted, which may be driven by their regular exposure to multiple hazards linked to COPD. For instance, several studies have observed positive associations between COPD incidence and mortality, and mining-related exposures, such as diesel engine exhaust, gypsum, and mineral dust.^42,43,49–51^ These findings are supported by a recent study of United States workers using Health and Retirement Study data.^43^ The study observed a greatly elevated risk of COPD among those in the mining industry after adjusting for known COPD risk factors, including smoking.^43^

### Silicosis, asbestosis, and idiopathic pulmonary fibrosis

Asbestosis and silicosis are diseases characterized by scarring of the lungs caused by exposure to asbestos and RCS, respectively.^52^ The elevated risk of silicosis observed among those in the mining industry was largely attributable to metal miners and those working in services incidental to mining. Specifically, gold miners experienced a substantially higher risk of silicosis than all other industry groups explored in this study. From 1974 to 1991, gold mines had the highest average surface-level RCS concentrations in Ontario compared to other ore-type mines, which may contribute to the elevated risk among workers in this group.^53^ During this period, the underground average RCS levels in Ontario gold mines were also above the exposure threshold set by the American Conference of Governmental Industrial Hygienists (ACGIH).^53^

Non-metal mining was also identified as a high-risk industry for asbestosis, which was driven by the asbestos miners within this group. The elevated risk among workers employed in the non-metal mining industry may also be linked to crude petroleum and natural gas industry. A 2007 study characterizing carcinogens in the Norwegian petroleum industry suggested that asbestos exposure may have occurred through maintenance work and asbestos-containing products prior to their elimination.^54^ The risk of asbestosis was also elevated in the pulp and paper industry, specifically among those working in pulp and paper mills, and miscellaneous paper converters. However, wide confidence intervals may suggest statistical imprecision. These findings support those of a 2004 IARC study that detected asbestos in air samples collected from several different departments of paper and paperboard mills across 12 countries.^7^ Due to its long latency period, elevated risks of asbestosis in these industries may be observed well beyond the implementation of exposure regulations and mine closures.^55^

This study also observed elevated risks of IPF across the forestry and logging, the pulp and paper, and all four mining industries. IPF is a similar respiratory disease to asbestosis and silicosis, but its cause is unknown.^56^ However, the American Thoracic Society and European Respiratory Society estimate that 26% of all IPF cases are associated with workplace exposures.^41^ Specific exposures include metal dust and mineral dusts (including RCS), organic dusts (including wood), and more broadly vapours, gases, dusts, and fumes.^41^

### Acute myocardial infarction

An elevated risk of AMI was observed among workers in each natural resource-based major-level industry. The findings of this study align with the conclusions of a review by the Nordic Expert Group for Criteria Documentation of Health Risks from Chemicals that assessed evidence of an association between several hazards and cardiovascular disease.^57^ This review observed limited and moderately convincing evidence of an association between cardiovascular disease and wood products industries, and the pulp and paper industry.^57^ Additionally, AMI has been associated with high exposures to several other occupational exposures common to the natural resource-based industries, including noise,^58^ diesel engine exhaust,^59^ and fine dust and particles.^57,60^ Specifically, the review observed strong evidence that asbestos and RCS exposure are associated with cardiovascular diseases, including AMIs.^57^ Past exposure to a fine aluminium dust known as McIntyre Powder, which was used from 1943 to 1979 in an attempt to prevent silicosis among Ontario miners, may also be associated with a slightly increased risk of AMI.^61^

### Carpal tunnel syndrome and Raynaud's phenomenon

CTS can be caused by repetitive, awkward, and forceful hand movements, vibration from hand-held tools, or mechanical stress on the palm.^22^ The risk of CTS was elevated across all major-level industries in this study, except for services incidental to mining. The findings of this study align with those of a recent Manitoba ODSS analysis, which observed a substantially increased risk of CTS among those in the paper and printing, and mining industries.^62^ Workers in natural resource-based industries, specifically logging and forestry services, and contract drillers, have high exposure to hand-arm vibration using motor-powdered equipment, which likely contributed to their higher susceptibility to CTS.^22,63^

RP can also be caused by exposure to hand-arm vibration, such as from hand-held power tools or pneumatic rock drills.^64^ It may also be associated with or exacerbated by cold.^65^ Approximately 80% of all compensated RP cases in the ODSS cohort are from the mining industry.^66^ This study observed considerably elevated risks of RP among specific groups in the mining industry, including gold and non-metal miners. Similarly, a 2025 study observed that the tools operated by German underground coal miners exposed workers to more vibration, although for shorter durations, compared to other blue-collar industries (Sun et al., 2025).^67^ Further, among Russian apatite miners, vibration exposure was linked to occupational disease outcomes even in working conditions that met industrial hygiene standards.^68^ Veneer and plywood workers, a specific group within the wood products industry, also exhibited an increased risk of RP. This increase could be linked to vibration exposure through machinery, although the extent and magnitude of the exposure has yet to be assessed.^69^

### Prevention

Advancements in industrial hygiene and new workplace safety policies over the last several decades have reduced detrimental occupational exposures.^7,27,63,70–72^ For example, increased ventilation and dust management strategies implemented in mines have lowered dust and radon exposures over time.^28,72,73^ Similar developments have been observed in the forestry and logging, wood products, and pulp and paper industries.^7,63^ Despite these improvements, workers across these industries continue to be exposed to many airborne and physical hazards.^23,70,74^ The elevated risks of several occupational diseases among all four industries indicate that early, targeted prevention efforts are necessary to protect workers from hazardous exposures. For instance, a recent study examining trends in RCS levels in Ontario mines from 2014 to 2018 observed concentrations in some mines exceeded the Ontario occupational exposure limit of 0.100 mg/m^3^ and the ACGIH threshold of 0.025 mg/m^3^.^70,75^ These conditions have remained relatively unchanged over multiple decades.^70^ Further, exposures below the ACGIH level may still pose a health risk to workers, as an evidence review by the Nordic Expert Group for Criteria Documentation of Health Risks from Chemicals observed an association between cardiovascular disease and 40 years of exposure to RCS at 0.01–0.02 mg/m^3^.^57^ Similarly, a 2022 study examining occupational disease risk among apatite miners observed harmful working conditions that fell short of industrial hygiene standards.^68^ Occupational diseases among apatite miners were often linked to workplace conditions characterized by heavy labour, vibration and noise exposure, and lapses in technological processes.^68^

Increasing cumulative and duration of exposure to some workplace hazards, such as RCS, are associated with an increasing risk of lung cancer.^76^ Further, workers in the natural resource-based industries are simultaneously exposed to more than one hazardous exposure. In 2024, a SYNERGY study observed that joint occupational exposure to several pairs of carcinogens linked to lung cancer were associated with a higher risk than each exposure separately.^77^ Taken together, gaining a deeper understanding of workplace hazards, and developing new initiatives to reduce occupational exposures is critical to reduce disease risks among workers in key natural resource-based industries.

### Limitations & strengths

This study has some limitations. Information on industry is reported at the time of the compensation claim, with no additional details on the duration of employment or job changes, which may contribute to some misclassification. Additionally, misclassification of disease status could occur as cases were identified from health records, which are subject to specific case definitions and data availability. Although administrative data is an effective source to identify occupational disease occurrence, there are some limitations. For example, the ODSS uses the ICD-9 diagnosis code 443 to define RP cases, which encompasses other peripheral vascular diseases.^78^ However, RP is more common than other peripheral vascular diseases captured by this code and has been associated with occupational exposures, unlike the other conditions.^21,66,79,80^ Secondly, due to limitations in the administrative data, this study could not include noise-induced hearing loss, despite it being one of the most compensated occupational diseases in Ontario.^8^ The study also lacks information on potential non-occupational confounders, such as ethnicity, education, income, and cigarette smoking status. Further, the ODSS cohort was created using accepted lost time claims from WSIB and does not capture those with unaccepted claims or workers who did not file a claim. Thus, the cohort may not represent all workers in Ontario and may underestimate the true risk of developing an occupational disease in the natural resource-based industries. There was also variability in follow-up time between cancer and non-cancer outcomes based on data availability, which should be considered when comparing risks across different outcomes.

This study also has several notable strengths. It leverages a large cohort of workers, providing sufficient statistical power to investigate a wide range of occupational diseases, unlike other studies. Importantly, it identifies significant health risks in key natural resource-based industries primarily located in Northern Ontario, which have been challenging to study in previous research.

## Conclusion

This study identifies several occupational diseases affecting workers in natural resource-based industries, underscoring the critical need to share up-to-date evidence to support worker health and safety. Despite improvements to health and safety practices over time, the findings of this study and previous studies examining occupational disease risks and workplace conditions suggest that there may remain gaps in prevention initiatives that leave these workers vulnerable to occupational disease. As such, this study highlights a need for early, targeted interventions aimed to reduce industry-specific hazardous physical, chemical, and biological exposures and promote worker safety. The findings can inform prevention strategies both within the workplace and in broader policy or public health contexts.

## Supplemental Material

sj-docx-1-wor-10.1177_10519815261476663 - Supplemental material for Occupational disease risks in natural resource-based industries among a large cohort of workers in Ontario, Canada Supplemental material, sj-docx-1-wor-10.1177_10519815261476663 for Occupational disease risks in natural resource-based industries among a large cohort of workers in Ontario, Canada by Taryn Thompson, Katie Goggins, Paul A Demers, Katherine J Jardine, Meg Parker and Jeavana Sritharan in WORK
