# Supplementary material for: Occupational disease risks in natural resource-based industries among a large cohort of workers in Ontario, Canada
Source: Work. 2026 Aug 21;85(2):583–96. doi: 10.1177/10519815261476663 (PMC13624341; doi:10.1177/10519815261476663)
Supplement: Supplementary file 1 [file sj-docx-1-wor-10.1177_10519815261476663.docx]

**Supplemental Material**

**Occupational disease risks in natural resource-based industries among a large cohort of workers in Ontario, Canada**

**Supplemental Table 1:** Diagnostic codes and case definitions used to identify occupational diseases in the Occupational Disease Surveillance System (ODSS).

| **Occupational disease** | **Years** | **Case definition** |
| --- | --- | --- |
| **Cancer** |  |  |
| Oral cavity and pharyngeal | 1964–2019 | ICD-O-3 sites C000–C148, excluding those with histology codes of 9050–9055, 9140, or 9590–9993. |
| Non-Hodgkin’s lymphoma | 1964–2019 | Nodal: ICD-O-3 sites C024, C098–C099, C111, C142, C379, C422, C770–C779 with the histology codes 9590–9597, 9670–9671, 9673, 9675, 9678–9680, 9684, 9687–9691, 9695, 9698–9702, 9705, 9708–9709, 9712, 9714–9719, 9724–9729, 9735, 9737–9738, 9811–9818, 9823, 9827 or 9837.  Extranodal: All ICD-O-3 sites except for C024, C098–C099, C111, C142, C379, C422, and C770–C779 with the histology codes 9590–9597, 9670–9671, 9673, 9675, 9678–9680, 9684, 9687–9691, 9695, 9698–9702, 9705, 9708–9709, 9712, 9714–9719, 9724–9729, 9735, 9737–9738.  All ICD-O-3 sites except for C024, C098–C099, C111, C142, C379, C420–C422, C424, and C770–C779 with the histology codes 9811–9819, 9823, 9827 or 9837. |
| Colorectal | 1964–2019 | ICD-O-3 sites C180–C189 or C260 (colon) or C199–C209 (rectum), excluding those with the histology codes 9050–9055, 9140 or 9590–9993. |
| Pancreatic | 1964–2019 | ICD-O-3 sites C250–C259, excluding those with the histology codes 9050–9055, 9140 or 9590–9993. |
| Nasal | 1964–2019 | ICD-O-3 sites C300–C319, excluding those with the histology codes 9050–9055, 9140 or 9590–9993. |
| Laryngeal | 1964–2019 | ICD-O-3 sites C320–C329, excluding those with the histology codes 9050–9055, 9140 or 9590–9993. |
| Lung | 1964–2019 | ICD-O-3 sites C340–C349, excluding those with the histology code of 9050–9055, 9140 or 9590–9993. |
| Leukaemia | 1964–2019 | ICD-O-3 sites C420, C421, and C424 (Aleukemic, sub leukemic and NOS, acute and chronic lymphocytic leukaemia), with the histology codes 9811–9819, 9823, 9827 or 9837. Included histology codes:   - 9826, 9835–9836 (acute lymphocytic leukaemia) - 9820, 9832–9834, 9940 (other lymphocytic leukaemia) - 9840, 9861, 9865–9867, 9869, 9871–9874, 9877–9879, 9895–9898, 9910–9912, 9920 (acute myeloid leukaemia) - 9891 (acute monocytic leukaemia) - 9863, 9875–9876, 9945–9946 (chronic myeloid leukaemia) - 9860, 9930 (other myeloid/monocytic leukaemia) - 9801, 9805–9809, 9931 (other acute leukemia) - 9733, 9742, 9800, 9831, 9870, 9948, 9963–9964 (aleukemic, subleukemic and NOS) |
| **Non-Cancer** |  |  |
| Carpal tunnel syndrome | 2002–2020  (3 years)* | At least 1 OHIP billing code of N290, which is associated with surgical median nerve decompression. |
| Acute myocardial infarction | 2007–2020  (1 year)* | At least 1 DAD or NACRS claim of ICD-10 I21. |
| Raynaud’s phenomenon | 2002–2020  (3 years)* | 2 or more OHIP claims of ICD-9 443. |
| Chronic obstructive pulmonary disease | 2006–2020 | At least 1 DAD (ICD-10 J41–J44), 3 OHIP (ICD-9 491–492, 496) or 3 NACRS (ICD-10 J41–J44) claims within a 2-year period. |
| Asthma | 2002–2020  (3 years)* | 2 or more OHIP claims of ICD-9 691 or 692 within 12 months. |
| Asbestosis | 2006–2020 | 2 or more OHIP claims of ICD-9 501x or NACRS J61 reports on a minimum of two different days, or at least one DAD J61 report. |
| Silicosis | 2006–2020 | 2 or more OHIP claims of ICD-9 502x or 2 NACRS J628 on a minimum of two different days, or at least one DAD report of J628. |
| Idiopathic pulmonary fibrosis | 2006–2020 | At least 1 DAD or 2 NACRS records of ICD-10 J841 or 2 OHIP claims of ICD-9 516. |

ICD, International Classification of Diseases; DAD, Discharge Abstract Database; NACRS, National Ambulatory Care Reporting System; OHIP, Ontario Health Insurance Plan

****look-back period applied.*

**Supplemental Table 2:** Risk of occupational diseases among workers in specific wood products industries, compared to all other workers in the ODSS cohort.

|  |  | **Sawmills, planning, & shingle mills** | | **Veneer and plywood** | | **Coffin & casket** | | **Sash, door & other millwork plants** | | **Wooden box & pallet operations** | | **Miscellaneous wood industries** | |
| --- | --- | --- | --- | --- | --- | --- | --- | --- | --- | --- | --- | --- | --- |
|  |  | Cases  (Workers) | HR  (95% CI) | Cases  (Workers) | HR  (95% CI) | Cases  (Workers) | HR  (95% CI) | Cases  (Workers) | HR  (95% CI) | Cases  (Workers) | HR  (95% CI) | Cases  (Workers) | HR  (95% CI) |
| **Cancer** |  |  |  |  |  |  |  |  |  |  |  |  |  |
| Oral cavity and pharyngeal |  | 36  (9,773) | 0.93  (0.67–1.28) | 9  (2,121) | 1.25  (0.65–2.40) | <6  (155) | - | 54  (18,376) | 0.91  (0.69–1.19) | 17  (3,715) | 1.62  (1.00–2.60) | 28  (6,587) | 1.25  (0.87–1.82) |
| Colorectal |  | 121  (9,775) | 1.02  (0.86–1.22) | 25  (2,120) | 1.01  (0.68–1.49) | <6  (155) | - | 169  (18,368) | 0.95  (0.81–1.10) | 40  (3,716) | 1.34  (0.98–1.82) | 66  (6,591) | 1.00  (0.78–1.27) |
| Pancreatic |  | 28  (9,777) | 1.21  (0.84–1.76) | 7  (2,121) | 1.41  (0.67–2.96) | <6  (155) | - | 28  (18,377) | 0.79  (0.54–1.14) | 8  (3,716) | 1.34  (0.67–2.69) | 10  (6,593) | 0.76  (0.41–1.41) |
| Laryngeal |  | 18  (9,777) | 1.30  (0.82–2.06) | <6  (2,121) | - | <6  (155) | - | 18 (18,377) | 0.93  (0.58–1.48) | <6  (3,716) | - | 10  (6,592) | 1.39  (0.75–2.59) |
| Lung |  | 209  (9,772) | 1.33  (1.16–1.52) | 39  (2,121) | 1.17  (0.85–1.60) | <6  (155) | - | 204  (18,376) | 0.87  (0.76–1.00) | 45  (3,716) | 1.17  (0.87–1.57) | 98  (6,590) | 1.14  (0.94–1.39) |
| Leukaemia & non-Hodgkin’s lymphoma |  | 69  (9,774) | 0.87  (0.68–1.10) | 26  (2,120) | 1.56  (1.06–2.29) | <6  (155) | - | 105  (18,362) | 0.84  (0.69–1.02) | 27  (3,715) | 1.25  (0.86–1.82) | 40  (6,592) | 0.85  (0.63–1.17) |
| **Non-Cancer** |  |  |  |  |  |  |  |  |  |  |  |  |  |
| Carpal tunnel syndrome |  | 42  (1,689) | 1.56  (1.15–2.11) | 15  (311) | 2.33  (1.41–3.87) | <6  (34) | - | 76  (5,328) | 0.92  (0.74–1.15) | 9  (1,044) | 0.67  (0.35–1.29) | 19  (1,147) | 0.90  (0.57–1.40) |
| Acute myocardial infarction |  | 452  (7,826) | 1.26  (1.15–1.38) | 91  (1,687) | 1.28  (1.04–1.57) | 6  (128) | 0.93  (0.42–2.08) | 616  (15,073) | 1.04  (0.96–1.13) | 134  (3,099) | 1.26  (1.07–1.50) | 248  (5,398) | 1.10  (0.97–1.25) |
| Raynaud’s syndrome |  | 19  (1,747) | 0.94  (0.60–1.48) | 10  (331) | 2.15  (1.16–4.00) | <6  (36) | - | 59  (5,441) | 0.99  (0.76–1.27) | 7  (1,063) | 0.79  (0.38–1.66) | 15  (1,175) | 0.99  (0.59–1.64) |
| Chronic obstructive pulmonary disease |  | 486  (7,699) | 1.37  (1.25–1.50) | 107  (1,653) | 1.39  (1.15–1.68) | 7  (127) | 1.01  (0.48–2.12) | 611  (14,899) | 1.03  (0.95–1.11) | 149  (3,045) | 1.45  (1.24–1.70) | 287  (5,339) | 1.26  (1.12–1.41) |
| Asthma |  | 32  (1,629) | 0.63  (0.44–0.89) | 7  (313) | 0.57  (0.27–1.21) | <6  (34) | - | 144  (5,051) | 0.98  (0.83–1.16) | 30  (991) | 1.20  (0.84–1.71) | 30  (1,101) | 0.79  (0.55–1.13) |
| Asbestosis |  | <6  (7,906) | - | <6  (1,700) | - | <6  (131) | - | 9  (15,171) | 0.66  (0.34–1.27) | <6  (3,127) | - | <6  (5,456) | - |
| Idiopathic pulmonary fibrosis |  | 23  (7,909) | 1.21  (0.80–1.83) | 7  (1,701) | 1.76  (0.84–3.69) | <6  (131) | - | 15  (15,175) | 0.52  (0.31–0.86) | 6  (3,129) | 1.27  (0.57–2.83) | 17  (5,457) | 1.57  (0.97–2.53) |

HR*, hazard ratio. Adjusted for birth year, sex, and age at start of follow up; CI, confidence interval; ODSS, occupational disease surveillance system.

**Supplemental Table 3:** Risk of occupational diseases among workers in specific pulp and paper industry groups compared to all other workers in the ODSS.

| **Occupational Disease** | **Pulp and paper mills** | | **Paper box and bag manufacturers** | | **Miscellaneous paper converters** | |
| --- | --- | --- | --- | --- | --- | --- |
|  | Cases  (Workers) | HR*  (95% CI) | Cases  (Workers) | HR*  (95% CI) | Cases  (Workers) | HR*  (95% CI) |
| **Cancer** |  |  |  |  |  |  |
| Oral cavity and pharyngeal | 33  (6,371) | 0.88  (0.63–1.25) | 38  (10,291) | 0.95  (0.69–1.30) | 32  (8,667) | 0.99  (0.70–1.40) |
| Colorectal | 147  (6,370) | 1.15  (0.97–1.35) | 130  (10,294) | 0.96  (0.81–1.14) | 136  (8,665) | 1.18  (1.00–1.40) |
| Pancreatic | 30  (6,372) | 1.21  (0.85–1.73) | 18  (10,297) | 0.66  (0.42–1.05) | 22  (8,669) | 0.94  (0.62–1.43) |
| Laryngeal | 11  (6.370) | 0.71  (0.39–1.29) | 16  (10,297) | 1.13  (0.69–1.85) | 15  (8,668) | 1.36  (0.82–2.25) |
| Lung | 167  (6,369) | 0.92  (0.79–1.08) | 198  (10,297) | 1.06  (0.92–1.22) | 179  (8,666) | 1.11  (0.96–1.28) |
| Leukaemia & non-Hodgkin’s lymphoma | 80  (6,366) | 1.01  (0.81–1.26) | 90  (10,291) | 1.00  (0.81–1.23) | 72  (8,667) | 0.94  (0.74–1.18) |
| **Non-cancer** |  |  |  |  |  |  |
| Carpal tunnel syndrome | 30  (581) | 2.66  (1.86–3.81) | 37  (1,553) | 1.23  (0.89–1.70) | 28  (1,284) | 1.06  (0.73–1.53) |
| Acute myocardial infarction | 368  (4,561) | 1.22  (1.10–1.35) | 415  (8,069) | 1.06  (0.96–1.17) | 336  (6,618) | 1.08  (0.97–1.20) |
| Raynaud’s phenomenon | 10  (632) | 0.85  (0.46–1.58) | 24  (1,596) | 1.14  (0.77–1.71) | 24  (1,324) | 1.32  (0.89–1.97) |
| Chronic obstructive pulmonary disease | 352  (4,486) | 1.15  (1.04–1.28) | 493  (7,961) | 1.18  (1.08–1.29) | 388  (6,498) | 1.12  (1.01–1.23) |
| Asthma | 17  (604) | 0.87  (0.54–1.41) | 64  (1,488) | 1.26  (0.99–1.62) | 36  (1,227) | 0.86  (0.62–1.19) |
| Asbestosis | 12  (4,646) | 1.41  (0.80–2.48) | 8  (8,164) | 0.84  (0.42–1.67) | 12  (6,693) | 1.61  (0.91–2.83) |
| Idiopathic pulmonary fibrosis | 26  (4,656) | 1.35  (0.91–1.98) | 29  (8,168) | 1.28  (0.89–1.84) | 24  (6,696) | 1.28  (0.86–1.91) |

HR*, hazard ratio. Adjusted for birth year, sex, and age at start of follow up; CI, confidence interval; ODSS, occupational disease surveillance system.
